# Characterization of mitochondrial genome and phylogenetic implications for Chinese black ant, *Polyrhachis dives* (Hymenoptera: Formicidae)

**DOI:** 10.1080/23802359.2017.1383204

**Published:** 2017-09-26

**Authors:** Jian-Hong Liu, Ping-Fan Jia, Jun-Qing Fu, Wen-Li Dan, Li-Ying Yang, Qing-Mei Wang, Zhang-Neng Li

**Affiliations:** a Key Laboratory of Forest Disaster Warning and Control of Yunnan Province, Southwest Forestry University, Kunming, China;; b School of Nursing and Rehabilitation, Xinyu University, Xinyu, China

**Keywords:** *Polyrhachis dives*, mitogenome, molecular phylogeny

## Abstract

Chinese black ant, *Polyrhachis dives* Smith, also known as *P. vicina* Roger has been used as a traditional edible insect and Chinese medicine in China and some southeast countries for thousands of years. In this study, Mitogenome of this species was assembled with high coverage using Illumina sequencing data and is 15,806 bp long in size. The base composition is 37.8% for A, 12.5% for C, 6.4% for G and 43.3% for T. The mitogenome contains 13 protein-coding genes, 22 transfer RNA genes and 2 ribosomal RNA genes. The phylogeny showed that it was closely related to *Camponotus atrox* (Hymenoptera: Formicidae) with high bootstrap value supported. The mitogenome of *P. dives* can provide essential DNA molecular data for further phylogenetic and evolutionary analysis.

Chinese black ant, *Polyrhachis dives* Smith, also known as *P. vicina* Roger is one of the members of the family Formicidae within the order Hymenoptera. *P. dives* is widely distributed in south China, India, Malaysia, Sri Lanka and Bangladesh. The ant has a complex social organization within the colonies (Tang et al. 1995). Chinese black ant has been used as a traditional edible insect in China and some southeast countries because it is rich in nutrients and has such healthcare functions as the regulation of immune system, the relaxation of fatigue and anti-aging (Cai et al. 1993). *P. dives* has also been used as Chinese medicine to treat diabetes, rheumatoid and osteoarthritis, inflammatory diseases, and central nerve system associated disorders for thousands of years (Huang and Xiao 2003).

The samples of *P. dives* were collected from Mengyang town (22.09°N, 100.90°E) of Jinghong City, Yunnan Province in southwest China. Specimen was deposited in Key Laboratory of Forest Disaster Warning and Control of Yunnan Province, Kunming, China (voucher no. KM20170326). Mitochondrial genomic DNA of Chinese black ant was extracted in accordance with the manufacturer’s instruction in the DNeasy Blood and Tissue kit (Qiagen, Hilden, Germany) for insect tissue. Genome shotgun reads were sequenced with the Illumina HiSeq platform (San Diego, CA) and complete mitochondrial genome was assembled with high coverage using Illumina sequencing data. The sequence was preliminarily aligned whithin the CLUSTAL X program in BioEdit software (Thompson et al. 1997; Hall 1999). Protein-coding genes (PCGs), rRNA and tRNA genes for *P. dives* were predicted by using MITOS tools (Bernt et al. 2013).

The complete mitogenome of *P. dives* is 15,806 bp long in size (GenBank MF919600). Nucleotide composition is 37.8% for A, 12.5% for C, 6.4% for G and 43.3% for T. Complete mitogenome of *P. dives* contains 13 protein-coding genes (PCGs), 22 transfer RNA genes, 2 ribosomal RNA genes and a major non-coding region known as the CR . Plus strand (J-strand) codes 9 PCGs (NAD2-3, NAD6, COX1-3, CYTB, ATP6 and ATP8) and 14 tRNAs, while the minus strand (N-strand) codes the other genes, including 4 PCGs (NAD1, NAD4, NAD4l and NAD5), 8 tRNAs and 2 rRNAs (16S rRNA and 12S rRNA). Gene arrangement of mitogenome for *P. dives* is identical to the most common type of the putative ancestor of insects (Boore 1999; Cameron 2014).

To validate the phylogenetic position for *P. dives*, the mitogenomes of 16 species in order Hymenoptera and an outgroup *Ceratitis capitata* (Diptera: Tephritidae) were clustered together with *P. dives* to construct ML tree by using the maximum likelihood method based on the Tamura-Nei model in MEGA 7 software (Tamura and Nei 1993; Kumar et al. 2016). The tree inferred from 500 replicates was taken to represent the phylogeny of the species analyzed in this study (Felsenstein 1985). Result indicates that *P. dives* is closely related to *Camponotus atrox* (Hymenoptera: Formicidae) with high bootstrap value supported (Figure 1). Furthermore, three clades were correctly identified as assigned and monophyletic family Vespidae, Apidae and Formicidae with high bootstrap confidence (Figure 1). In conclusion, the mitochondrial genome of *P. dives* deduced in present study can provide essential DNA molecular data for further phylogenetic and evolutionary analysis for order Hymenoptera.

**Figure 1.**
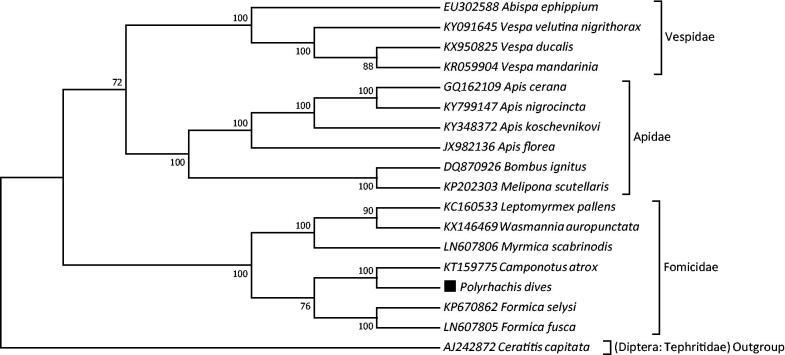
Molecular phylogeny of Chinese black ant, *Polyrhachis dives* (GenBank MF919600) and the related species in order Hymenoptera based on complete mitogenome. Phylogenic tree is constructed by maximum likelihood method with 500 bootstrap replicates. Genbank accession number for tree construction is listed before the scientific name of species. The position of *P. dives* was marked in solid square shape.

## References

[CIT0001] Bernt M, Donath A, Juhling F, Externbrink F, Florentz C, Fritzsch G, Putz J, Middendorf M, Stadler PF. 2013 MITOS: improved de novo metazoan mitochondrial genome annotation. Mol Phylogenet Evol. 69:313–319. 22982435 10.1016/j.ympev.2012.08.023

[CIT0002] Boore JL. 1999 Animal mitochondrial genomes. Nucleic Acids Res. 27:1767–1780. 10101183 10.1093/nar/27.8.1767 PMC148383

[CIT0003] Cai Y, Li AY, Xie PS, Zhao Y. 1993 Studies on the components of the ant and its preparation. Lishizhen Med Res. 4: 12–14. (In Chinese)

[CIT0004] Cameron SL. 2014 Insect mitochondrial genomics: implications for evolution and phylogeny. Annu Rev Entomol. 59:95–117. 24160435 10.1146/annurev-ento-011613-162007

[CIT0005] Felsenstein J. 1985 Confidence limits on phylogenies: An approach using the bootstrap. Evolution. 39:783–791. 28561359 10.1111/j.1558-5646.1985.tb00420.x

[CIT0006] Hall TA. 1999 BioEdit: a user-friendly biological sequence alignment editor and analysis program for Windows 95/98/NT. Nucleic Acids Symp Ser. 41:95–98.

[CIT0007] Huang NJ, Xiao SX. 2003 Study on *Polyrhachis vicina*. Res Pract Chin Med. 17:60–62. (In Chinese)

[CIT0008] Kumar S, Stecher G, Tamura K. 2016 MEGA7: molecular evolutionary genetics analysis Version 7.0 for bigger datasets. Mol Biol Evol. 33:1870–1874. 27004904 10.1093/molbev/msw054 PMC8210823

[CIT0009] Tamura K, Nei M. 1993 Estimation of the number of nucleotide substitutions in the control region of mitochondrial DNA in humans and chimpanzees. Mol Biol Evol. 10:512–526. 8336541 10.1093/oxfordjournals.molbev.a040023

[CIT0010] Tang J, Li S, Huang E, Zhang BY, Chen Y. 1995 Economic insect fauna of China Hymenoptera: Formicidae (1). Beijing, China: Science Press. (In Chinese)

[CIT0011] Thompson JD, Gibson TJ, Plewniak F, Jeanmougin F, Higgins DG. 1997 The clustal X Windows interface: flexible strategies for multiple sequence alignment aided by quality analysis tools. Nucleic Acids Res. 24:4876–4882. 10.1093/nar/25.24.4876 PMC147148 9396791

